# A Lupin (*Lupinus* *angustifolius*) Protein Hydrolysate Exerts Anxiolytic-Like Effects in Western Diet-Fed ApoE^−/−^ Mice

**DOI:** 10.3390/ijms23179828

**Published:** 2022-08-29

**Authors:** Guillermo Santos-Sánchez, Eduardo Ponce-España, Juan Carlos López, Nuria Álvarez-Sánchez, Ana Isabel Álvarez-López, Justo Pedroche, Francisco Millán, María Carmen Millán-Linares, Patricia Judith Lardone, Ignacio Bejarano, Ivan Cruz-Chamorro, Antonio Carrillo-Vico

**Affiliations:** 1 Instituto de Biomedicina de Sevilla, IBiS (Universidad de Sevilla, HUVR, Junta de Andalucía, CSIC), 41013 Seville, Spain; 2 Departamento de Bioquímica Médica y Biología Molecular e Inmunología, Facultad de Medicina, Universidad de Sevilla, 41009 Seville, Spain; 3 Departamento de Psicología Experimental, Universidad de Sevilla, 41009 Seville, Spain; 4 Department of Food & Health, Instituto de la Grasa, CSIC, Ctra, Utrera Km 1, 41013 Seville, Spain

**Keywords:** lupin, peptides, protein hydrolysates, anxiety, ApoE^−/−^, functional foods, peptidomics

## Abstract

Anxiety is the most prevalent psychiatric disorder worldwide, causing a substantial economic burden due to the associated healthcare costs. Given that commercial anxiolytic treatments may cause important side effects and have medical restrictions for prescription and high costs, the search for new natural and safer treatments is gaining attention. Since lupin protein hydrolysate (LPH) has been shown to be safe and exert anti-inflammatory and antioxidant effects, key risk factors for the anxiety process and memory impairment, we evaluated in this study the potential effects of LPH on anxiety and spatial memory in a Western diet (WD)-induced anxiety model in ApoE^−/−^ mice. We showed that 20.86% of the 278 identified LPH peptides have biological activity related to anxiolytic/analgesic effects; the principal motifs found were the following: VPL, PGP, YL, and GQ. Moreover, 14 weeks of intragastrical LPH treatment (100 mg/kg) restored the WD-induced anxiety effects, reestablishing the anxiety levels observed in the standard diet (SD)-fed mice since they spent less time in the anxiety zones of the elevated plus maze (EPM). Furthermore, a significant increase in the number of *head dips* was recorded in LPH-treated mice, which indicates a greater exploration capacity and less fear due to lower levels of anxiety. Interestingly, the LPH group showed similar thigmotaxis, a well-established indicator of animal anxiety and fear, to the SD group, counteracting the WD effect. This is the first study to show that LPH treatment has anxiolytic effects, pointing to LPH as a potential component of future nutritional therapies in patients with anxiety.

## 1. Introduction

Anxiety disorders (AnxDs), characterized by anxiety and fear, are the most common mental disorder worldwide [1]. They affect 33.7% of the global population during their lifetime, generating an important economic burden due to enormous healthcare expenditure [2]. AnxDs have serious consequences on physical and mental health (headache, irritability, breathing problems, depression, fatigue, etc.), affecting the course of normal daily activities of patients and reducing their quality of life [3]. Numerous studies have shown a strong relationship between anxiety and the consumption of diets rich in refined sugars and saturated fats [4,5]. Furthermore, the intake of these types of diet is the main risk factor for the generation of chronic diseases (diabetes, high blood pressure, cardiovascular diseases (CVDs), Alzheimer’s disease, and chronic obstructive pulmonary disease), which in turn have been shown to be closely related to the presence of anxiety [6,7,8,9,10,11]. Thus, anxiety is highly prevalent in patients with chronic diseases and can also increase the risk of worsening functional impairment, comorbidities, and mortality [12,13,14]. Within chronic diseases, there is a close connection between anxiety and memory loss. Thus, several pieces of evidence have shown how acute stress can hinder the memorization process [15,16]. For these reasons, anxiety is postulated to be a modifiable risk factor for chronic diseases.

Nutrition is considered a major risk factor for chronic diseases. Scientific evidence supports the view that diet changes have positive or negative effects on health [17]. Thus, fine control of the diet can be useful in preventing the onset of some diseases. In this regard, dietary supplementation has been considered a strategy to modulate different metabolic pathways [18,19]. In particular, nutritional psychiatry, based on diet improvement for the prevention and treatment of mental disorders, including anxiety, is gaining attention in the scientific community, which uses animal models to assess the influence of new nutritional strategies and pharmacological interventions [20].

There are several commercial treatments to reduce anxiety (selective serotonin reuptake inhibitors, barbiturates, benzodiazepines, analogues of benzodiazepine, etc.); however, many of them have important side effects that affect quality of life, such as drowsiness, sedation, confusion, and headache [21]. Therefore, the search for new natural and safer treatments has been of great interest over the last few years. The dietary supplementation with proteins and peptides have shown beneficial effects in human health modulating and/or optimizing several physiological processes and diseases such as hypertension, obesity, atherosclerosis, neurological dysfunctions, and other metabolic disorders [22,23,24,25,26,27]. There are many peptides from different foods that have also shown anxiolytic and antiamnesic activity [28]. Soymorphin-5 (YPFVV), soymorphin-6 (YPFVVN), and soymorphin-7 (YPFVVNA) [29], derived from soybean β-conglycinin, as well as rubiscolin-6 (YPLDLF) and rubimetide (MRW) [30], obtained from ribulose-1,5-bisphosphate carboxylase-oxygenase (RuBisCO) [31], have been shown to possess anxiolytic-like effects in mouse models. Moreover, ovolin (VYLPR) [32] from ovoalbumine, and peptides from αs1-casein [33,34] and β-lactoglobulin [35], have also been shown to reduce anxiety. Numerous peptides with antiamnesic effects from β-lactoglobulin have also been identified [36].

On the other hand, several studies have reported high levels of anxiety and spatial cognitive deficits (memory loss) in apolipoprotein E (ApoE) knockout mice (ApoE^−/−^) compared to wild-type mice [37,38,39]. ApoE deficiency results in an age-dependent dysregulation of the hypothalamic-pituitary-adrenal (HPA) axis through a mechanism that affects primarily the adrenal gland. The HPA axis regulates the secretion of glucocorticoids (GCs), which play important roles in several brain functions, including cognition. Dysregulation of the HPA axis has also been associated with behavioral alterations. Thus, ApoE^−/−^ mice show higher anxiety values than wild-type animals by using the elevated plus maze (EPM) test [40]. In addition, anxiety and memory loss can be accelerated and increased in ApoE^−/−^ fed a high-fat diet by oxidant and inflammatory effects [37,38]. Moreover, recent studies have shown a strong link between high cholesterol levels and anxiety [4,41].

Oxidative stress and inflammation play a key role in the anxiety process and memory impairment. In fact, alteration in redox balance, increased reactive oxygen species (ROS) production and high circulating inflammatory cytokines such as interleukin (IL)-1, IL-6, and tumor necrosis factor (TNF) have been detected both in anxiety patients and stressed animal models of anxiety [42,43,44]. In this line, our group has previously described that a *Lupinus angustifolius* protein hydrolysate (LPH) exerts hypocholesterolemic, anti-inflammatory and antioxidant effects in in vitro [45] and in vivo [46,47] models. In light of these considerations, this study aimed to identify LPH peptides with potential anxiolytic and antiamnesic effects and to evaluate the potential effects of LPH.

## 2. Results

### 2.1. Characterization of LPH

#### 2.1.1. Chemical Analysis of LPH

**Figure 1:**
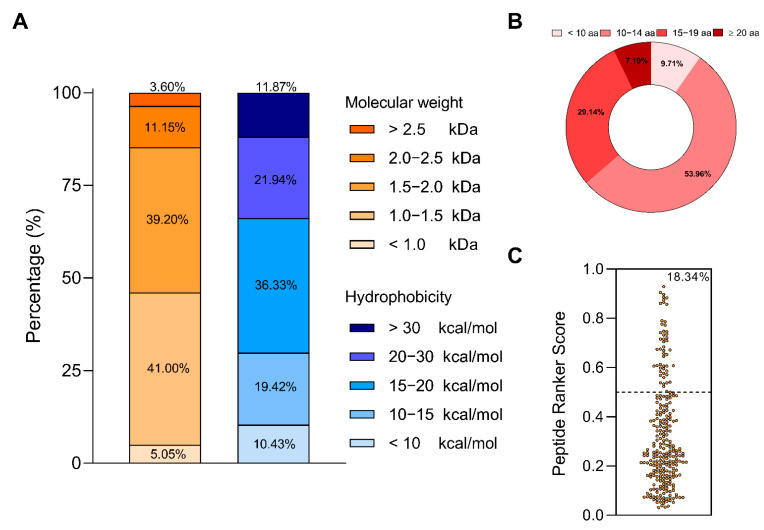
Percentage of molecular weight and hydrophobicity (**A**), length distribution (**B**), and predicted potential bioactivity (**C**) of the LPH peptides.

The molecular weights of the detected peptides ranged from 0.76 to 3.11 kDa. Specifically, the percentage of peptides identified with a molecular weight of <1 kDa, 1–1.5 kDa, 1.5–2.0 kDa, 2.0–2.5 kDa, and >2.5 kDa were 5.05%, 41.00%, 39.20%, 11.15%, and 3.60%, respectively (Figure 1A). LPH contained peptides with hydrophobicity <+10 kcal/mol (10.43%), +10–15 kcal/mol (19.42%), +15–20 kcal/mol (36.33%), +20–30 kcal/mol (21.94%), and >+30 kcal/mol (11.87%) (Figure 1A). Furthermore, the peptides consisted of 7–26 amino acid (aa) residues, being the most frequent peptides (83.10%) containing between 10–19 aa (Figure 1B). Regarding the aa composition, glutamic acid, leucine, and isoleucine were the most represented (12.90%, 10.30% and 8.00%, respectively), while tryptophan, and methionine were the least (0.4%, and 0.5%) (Table 1).

Finally, the bioactivity analysis of the LPH showed that 51 of the 278 sequences (18.34%) possess a score value greater than 0.5 threshold (Figure 1C).

#### 2.1.2. LPH Contains Peptides with Anxiolytic and Antiamnesic Effects

There were 278 peptides with an area greater than 107 identified in LPH (Supplementary Table S3). These peptides belonged mainly to conglutins, the main storage protein in lupin seed. Of the 278 identified sequences, 58 peptides (20.86%) with potential biological activity related to anxiolytic/analgesic effects were identified. In particular, 49 (17.62%) sequences contained a demonstrated antiamnesic motif and 9 (3.24%) sequences contained a demonstrated anxiolytic motif (Table 2).

**Figure 2:**
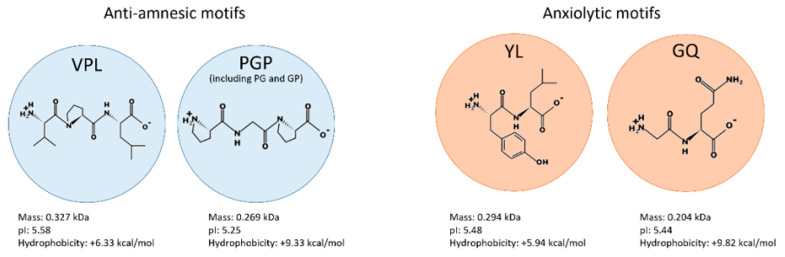
Physicochemical properties and primary structures of the anti-amnesic and anxiolytic motifs. pI, isoelectric point.

Of these 58 peptides, 41.38% were peptides from conglutin proteins, whereas 58.62% were from non-conglutin proteins. The tripeptides VPL and PGP, and the dipeptides PG and GP, were the sequences related to antiamnesic effects and identified with the BIOPEP-UWM IDs 3166, 3459, 3460, and 3461, respectively. The dipeptides YL and GQ were the sequences associated with anxiolytic effects and identified with the following IDs, 8310 and 2890, respectively. The physicochemical properties and primary structures of the identified motifs are shown in Figure 2.

### 2.2. In Vivo Experiments

#### 2.2.1. LPH Treatment Does Not Alter the Body Weight of Mice

To find the differences in weight changes between mice fed different diets and treated or not with LPH, the body weights of the mice were measured throughout the experiment. As shown in Table 3, there were no significant differences in the baseline body weight (BBW) at the beginning of the experiment among the experimental groups. Furthermore, after 16 weeks of diet, there were no differences in the final body weight (FBW) and in the body weight gain (BWG) between the groups fed WD and SD. In addition, 14 weeks of LPH treatment did not generate changes in the FBW and BWG of the mice, compared to the groups fed WD or SD.

#### 2.2.2. LPH Palliates the Anxious Effects Induced by WD Ingestion

**Figure 3:**
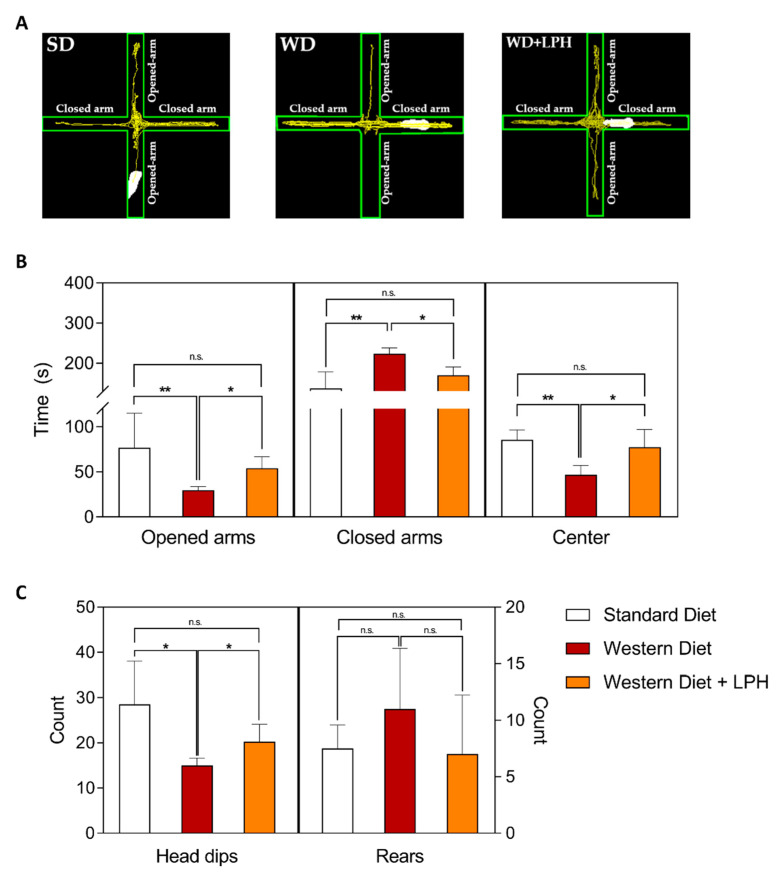
Representative images of the tracks of mice in the elevated plus maze (**A**). Time spent in opened arms, closed arms, and center zone (**B**), *head dips* and rears (**C**). Values are shown as the mean and standard deviation of each group. * *p* ≤ 0.05; ** *p* ≤ 0.01; n.s., not significant; SD, standard diet fed-mice; WD, Western diet-fed mice; WD + LPH, Western diet-fed mice treated with LPH; LPH, lupin protein hydrolysate.

As shown in Figure 3B, WD-fed mice spent significantly less time in the open arms of the elevated plus maze and more in the closed arms compared to the SD group. This effect was overcome by LPH treatment. Furthermore, the time spent in the center was significantly shorter in WD compared to SD and WD + LPH. Representative images of the tracks of the mice in EPM are shown in Figure 3A. Other anxiety-related behaviors, such as *head dips* and rears, were also evaluated. As shown in Figure 3C, the number of *head dips* was significantly lower in the WD group compared to the SD and WD + LPH groups, while no differences in the rears were observed among groups.

#### 2.2.3. LPH Treatment Does Not Improve Spatial Memory but Modulates WD-Induced Thigmotaxis, an Anxiety-Related Behavior

**Figure 4:**
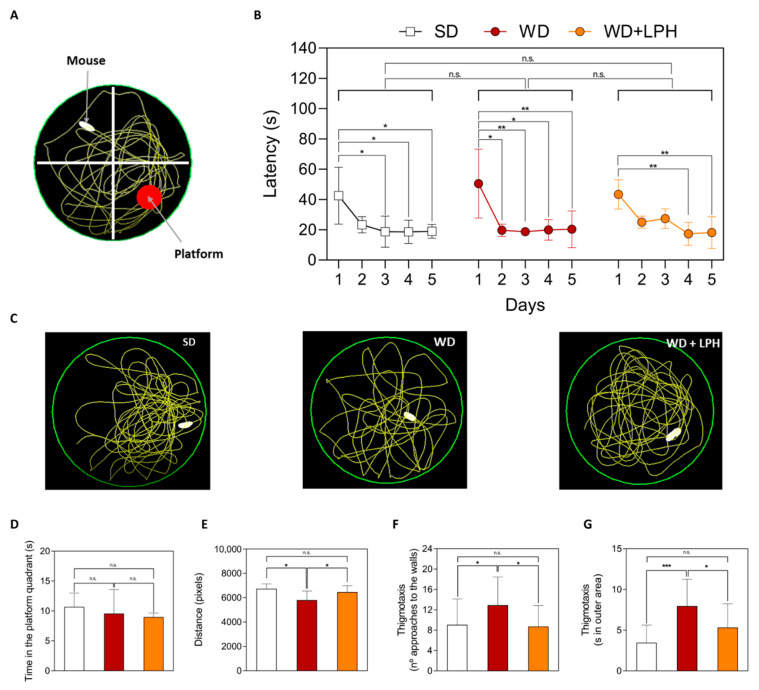
Representative image of the acquisition phase of the Morris water maze (**A**); latency of the mice during the five days (**B**). Representative images of the trial phase (**C**); time in the platform zone (**D**), distance traveled (**E**) and thigmotaxis (**F**,**G**). Values are shown as the mean and standard deviation of each group. * *p* ≤ 0.05; ** *p* ≤ 0.01; *** *p* ≤ 0.001; n.s., not significant; SD, standard diet-fed mice; WD, Western diet-fed mice; WD + LPH, Western diet-fed mice treated with LPH; LPH, lupine protein hydrolysate.

To study spatial learning and memory, the platform in the Morris water maze (MWM) was placed according to the Figure 4A. During nonvisible platform sessions (days 1–5), all groups learned to reach the submerged platform, due to the decrease in the mean latency over the consecutive five days of the learning period in all groups (Figure 4B). There were no significant differences between the groups in the latency time. After the removal of the platform (trial phase), there were also no differences in the time spent in the platform zone among the groups (Figure 4D), but curiously, there was a decrease in the total distance traveled for the WD-fed mice compared to the SD-fed mice (Figure 4E). This effect was overcome by the LPH treatment. Moreover, thigmotaxis was significantly higher in the WD diet group compared to the SD group, while LPH was able to reverse this increase, reducing thigmotaxis to values significantly different to the WD group, both considering the number of times animals approached the walls of the pool (Figure 4F) and the time spent in the outer area of the pool (Figure 4G). Representative images of the tracks of mice in the trial phase are shown in Figure 4C.

## 3. Discussion

LPH is a mixture of low molecular weight peptides obtained after hydrolysis of *L. angustifolius* proteins with Alcalase^®^, which have shown beneficial effects on oxidant and inflammatory status in different models [45,46,47]. Due to inflammation and oxidative stress are key processes in anxiety and memory impairment, the present work aimed to study the potential anxiolytic and antiamnesic effects of LPH. To achieve this goal, a multidisciplinary study has been conducted using a combination of analytical, molecular, biochemical, and behavioral techniques.

Since the bioactivity of food-derived peptides depends on their physicochemical features, such as length, hydrophobicity, and amino acid sequence, our first objective was to identify the composition of LPH peptides. Mass spectrometry analysis revealed the presence of 278 sequences from the *L. angustifolius* database in LPH, of which 58% derive from conglutins, the main seed storage proteins in lupin [53]. Furthermore, we found that approximately 20% of LPH peptides are potentially bioactive. In addition, the physical-chemical analysis showed that LPH mainly contains small-sized and hydrophobic peptides. Both are important features of peptides that determine their interaction with several physiological targets and their bioactivities, and these factors have also been demonstrated to influence peptide self-assembly, emulsifying capacity, and other properties, including biostability and potentially bioavailability [54].

The peptides analysis allowed us to identify sequences containing some known anxiolytic and antiamnesic motifs. Specifically, we identified 4 peptides (VPL, PGP, PG, and GP) present in 49 different sequences with antiamnesic effects and 2 peptides (YL and GQ) in 9 different sequences with anxiolytic effects. LPH contained 5 different sequences that present the YL dipeptide, which is able to activate the 5-hydroxytryptamine (serotonin) receptor 1A, the dopamine D1 receptor, and the type A receptor of c-amino butyric acid in mice, which play a pivotal role in anxiety. On the other hand, dipeptide YL has shown comparable effects to diazepam in equal doses [50], while PGP, PG, and GP have been demonstrated to enhance memory consolidation processes in the central nervous system [49]. In accordance with these data, the present study reports the beneficial effects of 14 weeks of LPH treatment on WD consumption-induced anxiety in ApoE^−/−^ mice. In fact, WD-fed ApoE^−/−^ mice have previously been demonstrated to successfully reproduce spatial cognitive deficits (memory loss) and anxiety status through a dysregulation of the HPA axis that regulates GCs synthesis, which plays an important role in several brain functions [38,39]. LPH exhibited anxiolytic-like activity, with no differences in learning or spatial memory, and its effects were not related to change in body weight, since mice belonging to different groups did not show a significant difference in BWG.

It is well known that high-fat and high-free-sugar diets are part of the environmental factors that can aggravate or favor the development of anxiety [55,56]; many reports have shown that a high-fat diet accelerates cognitive deficits and anxiety in ApoE^−/−^ mice [37]. To study anxiety, we used the EPM, a well-established test to evaluate anxiolytic/anxiety-like behaviors. In the EPM, mice experience the natural conflict between exploring a new place and the tendency to avoid a dangerous area [57]. We observed that WD significantly increases anxiety behavior since WD-fed mice remained less time on the opened arms and the center of the platform in the EPM compared to SD-fed mice. Opened arms and center areas are considered anxiety zones because rodents have an innate fear of elevated open spaces and tend to spend less time in them [58,59]. Thus, mice treated with anxiolytic drugs (i.e., diazepam) remained longer in the opened arms and in the center of the EPM [60]. Interestingly, WD-fed mice treated with LPH remained longer in the opened arms and in the center zone, and less time in the closed arms compared to the WD group. In addition, mice fed with WD showed fewer *head dips* in comparison to the control group. This behavior, which consists of lowering the head over the sides of the opened arms toward the floor, is considered exploratory and is related to a lower level of anxiety and fear [61]. These results are consistent with previous studies in humans [62] and mice [63], in which the anxiogenic power of a high-fat diet is also demonstrated. Interestingly, a significant increase in the number of *head dips* was recorded in LPH-treated mice, pointing to a higher exploration capacity and less fear, all caused by lower levels of anxiety.

The results obtained in the MWM revealed no impairment in memory or spatial learning after WD consumption. There were no differences in latency time or time spent in targeted section between mice fed with SD and WD. This fact could be associated with the age of the mice and the time of WD consumption. Janssen et al. concluded that ApoE^−/−^ mice perform MWM with better results than wild-type ones and demonstrated that WD does not alter the results in ApoE^−/−^ mice [64]. Furthermore, Champagne et al. showed that older ApoE^−/−^ mice obtain the worst results in MWM [65]. Apart from that, the present study shows that WD-fed mice covered less distance than mice from SD and LPH groups. Several pieces of evidence have shown that changes in distance may be due to alterations in the motivation to find the platform and greater capacity for exploration [66], but also due to lower activity or worse fitness [67]. Furthermore, WD-treated mice exhibited more *thigmotaxis* than the LPH group. Moreover, LPH-treated mice showed similar *thigmotaxis* to the SD group. This behavior is a well-established indicator of animal anxiety and fear [68,69]. This fact is consistent with the results observed in the EPM, strengthening the protective effect of LPH on WD-induced anxiety.

Although bioactive peptides from white eggs [70], salmon [71], bovine casein [72], or soy [29] have been described to exert anti-anxiety activity, to our knowledge, this is the first study to report the anxiolytic-like properties of a protein hydrolysate from lupin. High levels of oxidative stress and inflammation in the brain have been widely reported to be two of the main contributing factors involved in the development of anxiety [42,43,44]. Moreover, recent studies have shown a strong link between high cholesterol levels and anxiety [69]. Our group has previously shown that LPH exerts anti-inflammatory, antioxidant, and lipid-lowering effects both in ApoE^−/−^ mice [47,73] and humans [45,46]. Therefore, we suggest that these LPH properties may also be directly or indirectly responsible for the anxiolytic-like effects. In addition, the presence of peptides in the LPH with already demonstrated anxiolytic effects similar to those of diazepam, such as YL and GQ, could also be the cause of the demonstrated anxiolytic effects. However, the presence of other peptides in the LPH that have not yet proved their anxiolytic effects cannot be ruled out.

As in each study, this has certain solvable limitations. The number of mice used was limited (*n* = 4 per group); however, i) a small number of mice was sufficient to achieve significant differences, ii) two different anxiety analyses were performed to confirm the effect, and iii) the Cohen’s test analysis shows a large size effect on each variable studied (Supplementary Table S2). We also consider important to highlight that an SD + LPH group has not been included in the study, since SD mice do not exhibit anxious behaviors. In fact, the only reason we used an SD group was to check that WD consumption generates anxiety.

The main strength of this work is the multidisciplinary strategy used. First, a detailed chemical characterization of the LPH composition was performed by using nano-HPLC-MS/MS and UHPLC-HRMS to identify its peptide composition. Afterward, an in silico study was carried out for the identification of anxiolytic and antiamnesic peptides. Finally, an in vivo study confirmed through two different tests (EPM and thigmotaxis during the MWM) that LPH treatment palliates the anxious effects generated by the ingestion of WD. This study is the first to show the in vivo anxiolytic-like effect of a plant-derived total protein hydrolysate.

## 4. Materials and Methods

### 4.1. LPH Preparation

LPH was produced at the Instituto de la Grasa (CSIC, Seville, Spain), as previously described [45]. Briefly, the lupin protein isolate was resuspended in distilled water (10% *w*/*v*) and hydrolyzed in a bioreactor at pH 8 and temperature 50 °C using Alcalase^®^ 2.4 L (2.4 AU/g; Novozymes, Bagsvaerd, Denmark) for 15 min. The enzyme was inactivated by heating at 85 °C for 15 min; after centrifugation at 8000 rpm for 15 min, the supernatant containing LPH was collected and lyophilized. Finally, it was dissolved in 0.9% saline solution to obtain the LPH necessary for the duration of the experiment, filtered, autoclaved, aliquoted, and stored at −80 °C. The chemical stability and characterization of LPH were checked out at the several steps of this process through HPLC, no differences were observed (data not shown).

### 4.2. Purification and Concentration of Peptides

An amount of 1 mg of LPH was acidified with aqueous trifluoroacetic acid (TFA) at pH 2.5, loaded into the Bond Elut C18 EWP cartridge (Aligent, Santa Clara, CA, USA) (previously washed with acetonitrile (ACN) and conditioned with 0.1% TFA), and washed with 3 mL of 0.1% TFA. The elution was carried out with 0.5 mL ACN/H_2_O (50:50, *v/v*) containing 0.1% TFA, and the peptides were dried in a Speed Vac SC250 Express (Thermo Savant, Holbrook, NT, USA). The dry residue was reconstituted in 150 µL of 0.1% formic acid in H_2_O.

### 4.3. Peptides’ Analysis and Identification by Mass Spectrometry

The peptides were studied by nano-HPLC using an Ultimate 3000 coupled to an Orbitrap Elite mass spectrometer (Thermo Fisher Scientific, Bremen, Germany), as previously described [74]. The preconcentration of the samples (20 µL) was performed on a µ-precolumn (Thermo, 300 µm i.d. 5 mm Acclaim PepMap 100 C18, 5 µm particle size, 100 Å pore size) using H_2_O/ACN (99:1 *v*/*v*) with 0.1% TFA (*v*/*v*) at a flow rate of 10 µL/min. The peptides were dispersed on an EASY-Spray column (Thermo, 15 cm × 75 µm i.d. PepMap C18, 3 µm particles, 100 Å pore size).

The peptide spectra were obtained using the same parameters described in our previous work [73]. The protein sequence database of *L. angustifolius* (31,386 sequences) was downloaded from UnitProt and used for the identification of raw data spectra using Proteome Discoverer v1.3 (Thermo) in combination with the Mascot search engine v2.3.02. Precursor ion tolerance and the fragment ion tolerance were 10 ppm and 0.05 Da, respectively; no enzyme was used for digestion and methionine oxidation was considered as dynamic modification. The decoy function, set at 1%, was used for false discovery rate calculations.

### 4.4. Bioactivities Peptide Analysis

The physicochemical properties (molecular weight, amino acid composition, and hydrophobicity) of the peptides were obtained using the open access ProtParam tool (https://web.expasy.org/protparam/, accessed on 1 April 2022) [75]. The peptide Ranker tool (http://distilldeep.ucd.ie/PeptideRanker/, accessed on 1 April 2022) was used to predict the bioactivity of LPH [76]. It provides scores in the range of 0−1, being 1 the most active. The threshold was fixed at 0.5; therefore, peptides with scores above 0.5 were labeled as ‘bioactive’. To identify sequences with demonstrated bioactive motifs, the peptides were analyzed using the BIOPEP-UWM database (http://www.uwm.edu.pl/biochemia/index.php/pl/biopep/, accessed on 1 April 2022) [52]. In addition, the primary structure of the motifs was drawn using the PepDraw tool (https://pepdraw.com/, accessed on 1 June 2022).

### 4.5. Animals and Experimental Design

The experimental design is shown in Supplementary Figure S1. Twelve male ApoE^−/−^ mice (B6.129P2-ApoEtm1Unc/J) were housed in the animal facility of the Faculty of Psychology (University of Seville, Seville, Spain) under specific pathogen-free conditions in a room with controlled temperature (22 ± 2 °C), humidity (<55%), and a 12-h light–dark cycle with free access to water and food. The mice were housed in a sealsafe^®^ 1285L cage (Tecniplast, Italy) [77] with a floor area of 542 cm^2^ and a maximum air speed at the animal level of 0.05 m/s. Four mice were housed per cage. The particular characteristics of these cages allow no air drafts at the animal level, avoiding the risk of stress and heat loss. The animals were initially classified into two groups: mice fed a standard diet (SD, *n* = 4, Teklad Global 14% Protein Rodent Maintenance Diet, ENVIGO, Indianapolis, IN, USA) [78] and mice fed a Western diet (WD, *n* = 8, 58V8-45 kcal% fat, TestDiet, St. Louis, MO, USA) [79] from the Special Diets Production Section of the University of Granada (Granada, Spain). The composition of each diet is specified in Supplementary Table S1.

Six-week-old mice from the WD group were randomly divided into two groups and treated intragastrically with LPH (100 mg/kg, *n* = 4) or vehicle (*n* = 4) for 14 weeks, respectively. Thus, the experimental groups were set as follows: SD-fed mice group (SD, *n* = 4), WD-fed group (WD, *n* = 4), and WD-fed and LPH-treated (100 mg/kg) mice group (WD + LPH, *n* = 4). SD-fed mice were also intragastrically treated with vehicle. The dose of LPH was selected based on our previous studies [45,46,47,73]. Individual body weight was measured and recorded weekly. Behavioral tests were performed at the Laboratory of Animal Behavior & Neuroscience (a specific installation inside the Animal Facility of the Faculty of Psychology), where the animals were placed a week earlier for their habituation. The tests were carried out with a 10-day inter-test interval.

The experimental procedures were approved by the Ethics Committee of the Virgen Macarena-Virgen del Rocío University Hospital (reference number 21/06/2016/105) and were carried out under Spanish legislation and the EU Directive 2010/63/EU for animal experiments.

### 4.6. Behavioral Tests

#### 4.6.1. Elevated Plus Maze

**Figure 5:**
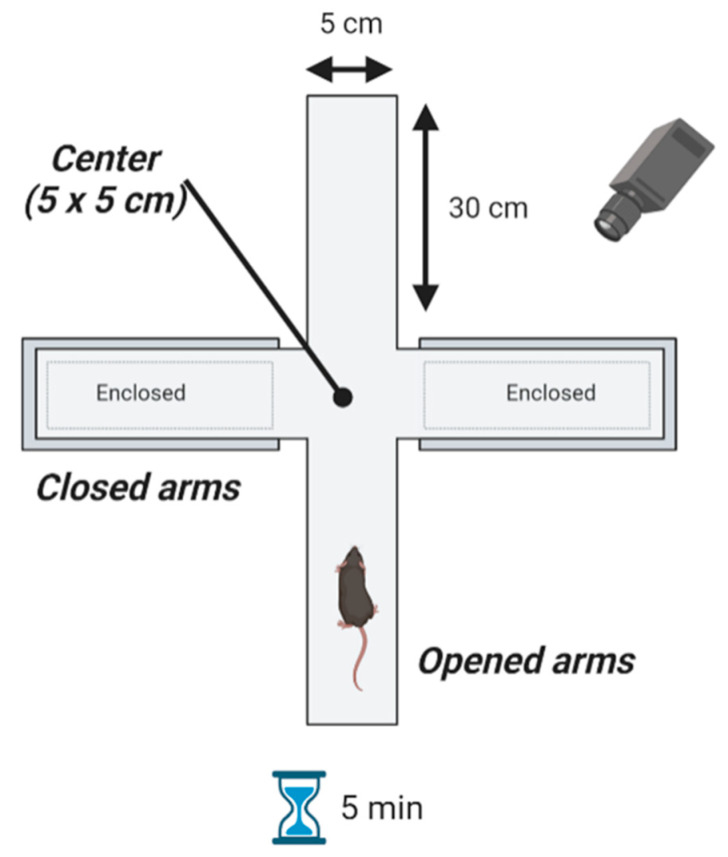
Characteristics of the Elevated Plus Maze. The maze consists of four arms: two non-consecutive open arms (30 cm long × 5 cm wide) and two closed arms that generate a common center zone (5 × 5 cm). The EPM was placed 60 cm above the floor. To start the test, each mouse was placed in one of the opened arms facing the opposite direction of the center and was free to move for 5 min. All sessions were recorded using a camera located over the maze. Figure created by BioRender.com.

Anxiety-like behavior was evaluated using the EPM test. It was performed as previously described [32]. Briefly, the maze consists of four arms made out of polyvinyl chloride; two non-consecutive opened arms (30 cm long × 5 cm wide) and two closed arms that generate a common center zone (5 × 5 cm). The EPM was placed 60 cm above the floor in the center of a room (286 × 288 × 320 cm; w-l-h respectively) illuminated by four 100-W halogen lamps. The characteristics of the EPM are shown in Figure 5. In order to minimize exploratory behavior and facilitate habituation to the context, mice were placed in the room for 45 min prior to the test. To start the test, each mouse was placed in one of the opened arms facing the opposite direction to the center and was free to move for 5 min. All sessions were recorded using a camera located over the maze. For the trials, the experimenter remained in an adjoining zone to control the video tracking system. Additionally, the observer could see the performance of the animal in real time on a monitor. Other anxiety-related behaviors, such as *head dips* and rears, and the number of times that mice showed them, were also annotated and recorded. The test started at 11:30 a.m. during the light phase of the light-dark cycle, and none of the researchers stayed in the room while the test took place. The floor of the elevated plus maze apparatus was cleaned with 10% ethanol between tests. Subsequently, the recording was processed using the Animal Tracker plugin for ImageJ v. 1.53k software (National Institutes of Health-NIH-, Bethesda, MD, USA) and the time spent in the arms and center of the maze was measured. Opened arms and center areas are considered anxiety zones according to [58,59]. Analyses were carried out under blind conditions by three investigators. Representative videos are available in Videos S1–S3.

#### 4.6.2. Morris Water Maze

**Figure 6:**
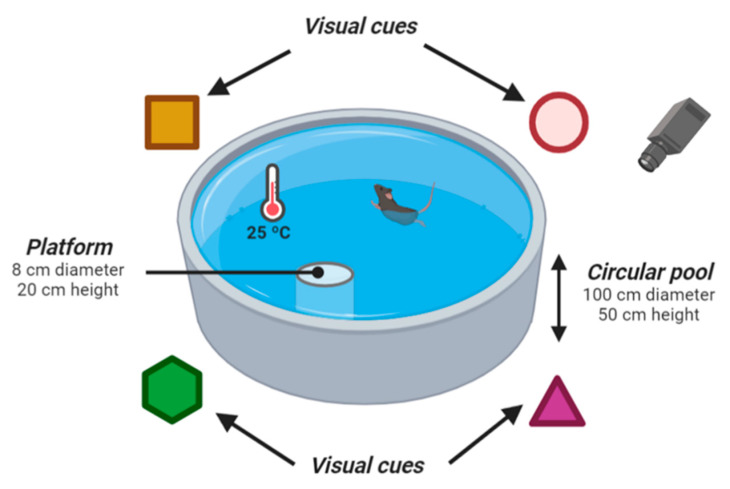
Characteristics of the Morris Water Maze. The test consists of a circular pool (100 cm in diameter) filled with water (at 25 °C) and a circular platform (8 cm in diameter, 20 cm in height) placed in a specific zone of the pool. The pool was virtually divided into four different quadrants and different visual clues were located on the walls of the room. All sessions were recorded with a video monitoring system that overlooks the pool from above. Figure created by BioRender.com.

The MWM was designed as a method to study spatial memory and learning processes. The experimental procedures were performed as described by Janseen et al. [64]. Briefly, the test consists of a circular pool (100 cm in diameter) filled with water (at 25 °C) and a circular platform (8 cm in diameter, 20 cm in height) located in a specific quadrant of the pool. It was virtually divided into four different sections, and different visual clues were located on the walls of the room (characteristics of the MWM are shown in Figure 6). The test was carried out for 5 consecutive days. To avoid the use of possible intramaze cues to solve the task, the experimental apparatus was randomly rotated between sessions. On day 0, mice received two habituation trainings; animals were located in two different sections and allowed to swim for 90 s until they reached the visible platform (2 cm above the water surface). Once on the platform, the mice stand there for 15 s. On days 1–5, animals were placed in each section and allowed to swim for 90 s or until they reached the non-visible platform. In this phase, the water was opaque by adding a white dye (lime) and the time between tests was 45 min. Finally, on the fifth day, the platform was removed, and the mice were placed in the pool for 90 s (the scheme of the Morris Water Maze protocol is shown in Supplementary Figure S2). All sessions were recorded with a video tracking system that overlooked the pool from above. The test started at 11:30 a.m. during the light phase of the light–dark cycle, and the experimenter stayed in an adjoining zone for the test. The latency time, distance traveled, and time spent in each quadrant were analyzed using the Animal Tracker plugin for ImageJ software (NIH). In addition, *thigmotaxis*, considered as the times the animal approaches the walls of the pool and the time spent in the outer area (15% of the apparatus) of the pool, was calculated. Analyses were carried out under blind conditions by three investigators. Representative videos are available in Videos S4–S6.

### 4.7. Statistical Analysis

All results were presented as mean ± standard deviation, and the statistical analysis was carried out using one-way ANOVA followed by Dunn’s post hoc test using Jeffreys’s Amazing Statistics Program (JASP v. 0.16.3, Amsterdam, The Netherlands). A difference with a *p*-value ≤ 0.05 was considered statistically significant. The size effect was analyzed using Cohen’s test, and a *d*-value > 0.80 was considered as ‘large effect size’.

## 5. Conclusions

In conclusion, this is the first study to show the in vivo anxiolytic effects of a lupin protein hydrolysate. Moreover, several sequences containing peptide motifs associated with anxiolytic effects were identified within the LPH mixture. Future studies will be needed to investigate the molecular mechanisms that cause the anxiolytic effect of LPH, as well as to compare this effect with an anxiolytic drug such as diazepam. In addition, several strategies, such as the incorporation of peptides into biocompatible vehicles to enhance their stability and bioavailability during transepithelial transport, are recommended for future investigation. The present study confirms the pleiotropic effects of the peptide mixture, including anxiolytic effects, pointing to LPH as a potential component of future nutritional therapies in patients with anxiety, being a possible strategy to reduce the consumption of drugs with side effects.

## Figures and Tables

**Table 1:** Amino acid composition of the identified peptides in the LPH.

Amino Acid	No.	%
Glu (E)	493	12.9
Leu (L)	396	10.3
Ile (I)	307	8.0
Pro (P)	307	8.0
Arg (R)	287	7.5
Asp (D)	275	7.2
Val (V)	249	6.5
Gly (G)	224	5.8
Ser (S)	202	5.3
Gln (Q)	196	5.1
Asn (N)	185	4.8
Lys (K)	151	3.9
Ala (A)	130	3.4
Thr (T)	130	3.4
Phe (F)	122	3.2
Tyr (Y)	84	2.2
His (H)	60	1.6
Trp (W)	19	0.5
Met (M)	16	0.4
Cys (C)	0	0.0

Ala, alanine; Arg, arginine; Asn, asparagine; Asp, aspartic acid; Cys, cysteine; Gln, glutamine; Glu, glutamic acid; Gly, glycine; His, histidine; Ile, isoleucine; Leu, leucine; Lys, lysine; Met, methionine; Phe, phenylalanine; Pro, proline; Ser, serine; Thr, threonine; Trp, tryptophan; Tyr, tyrosine; Val, valine.

**Table 2:** The number of identified LPH peptides with anti-amnesic and anxiolytic activity.

Effect	Bioactive Peptide Motif ^a^	BIOPEP-UWM ID ^b^	Origin Protein ^c^	Accession Number ^c^	N. Peptides	Reference
anti-amnesic	VPL	3166	Non-conglutin proteins		1	[48]
	PGP	3459	α-Conglutin	F5B8V7	3	[49]
	PG	3460				
	GP	3461				
			β-Conglutin	F5B8W1	14	
				F5B8W2		
				F5B8W3		
			Non-conglutin proteins		31	
anxiolytic	YL	8310	α-Conglutin	F5B8V6	4	[50]
			Non-conglutin proteins		1	
	GQ	2890	α-Conglutin	F5B8V6	3	[51]
				F5B8V7		
			Non-conglutin proteins		1	
TOTAL					58	

^a^ 1-letter amino acid code. ^b^ ID number present in the BIOPEP-UWM database [52]. ^c^ Accession number present in “UniProtKB” (http://www.uniprot.org/, accessed on 1 April 2022).

**Table 3:** Body weight parameters.

Parameter (g)	Experimental Group		
	SD	WD	WD + LPH
BBW	20.35 ± 0.41	20.98 ± 0.36	20.88 ± 0.49
FBW	26.20 ± 0.87	26.50 ± 0.54	27.15 ± 0.69
BWG	5.85 ± 1.18	5.53 ± 0.65	6.28 ± 1.09

Baseline body weight (BBW), final body weight (FBW) and body weight gain (BWG) in ApoE^−/−^ mice. Values are shown as the mean and standard error of the mean of each group. SD, standard diet fed-mice; WD, Western diet-fed mice; WD + LPH, Western diet-fed mice treated with LPH. No statistical differences were observed between the groups for each weight parameter.

## Data Availability

Data are contained within the article and Supplementary Materials.

## Supplementary Materials

The supporting information can be downloaded at: www.mdpi.com/article/10.3390/ijms23179828/s1.
