# Supplementary material for: A Lupin (Lupinus angustifolius) Protein Hydrolysate Exerts Anxiolytic-Like Effects in Western Diet-Fed ApoE−/− Mice
Source: Int J Mol Sci. 2022 Aug 29;23(17):9828. doi: 10.3390/ijms23179828 (PMC9456304; doi:10.3390/ijms23179828)
Supplement: Supplementary file 1 [file ijms-23-09828-s001.zip › ijms-1857643-supplementary/ijms-1857643-supplementary material.pdf]

Table S1. Macronutrient's composition of diets

Macronutrients	Diet	
	SD	WD
Fat (% of energy)	13.0	46.1
Carbohydrates (% of energy)	67.0	35.8
Protein (% of energy)	20.0	18.1
Total energy (kcal/g)	2.90	4.60

Energy provided from the different macromolecules in both diets. The complete nutritional description is available in [1] for SD, and in [2] for WD. SD, Standard diet; WD, Western diet.

Table S2. Statistical analysis of different parameters evaluated in this study.

Parameter	ANOVA	F-value	SD vs WD		WD vs WD + LPH		SD vs WD + LPH	
			p value	Size effect	*p* value	Size effect	*p* value	Size effect
Opened arms	0.047	4.37	0.005	2.090	0.015	-1.056	0.347	1.034
Closed arms	0.006	9.79	0.002	-3.094	0.031	1.953	0.163	-1.141
Center	0.008	8.49	0.004	2.769	0.020	-2.170	0.278	0.600
Head dips	0.033	5.09	0.002	2.237	0.038	-0.870	0.119	1.367
Rears	0.424	0.95	0.188	-0.780	0.151	0.892	0.441	0.111
Distance	< 0.001	11.16	< 0.001	1.623	0.009	-1.152	0.136	0.471
Thigmotaxis (nº approaches to the wall)	0.040	3.48	0.025	-0.781	0.021	0.848	0.470	0.067
Thigmotaxis (s)	0.002	7.40	< 0.001	-1.601	0.045	0.937	0.051	-0.664
Time in platform zone	0.679	0.41	0.403	0.411	0.129	0.215	0.085	0.626

p-value show the statistical analysis between groups using the Dunn’s pos-hoc test. Size effect was carried out by the Cohen's test. LPH, lupin protein hydrolysate; SD, standard diet; WD, western diet.

Figure S1. Schematic diagram of the experimental design of the study showing mice groups, dietary, and intervention.

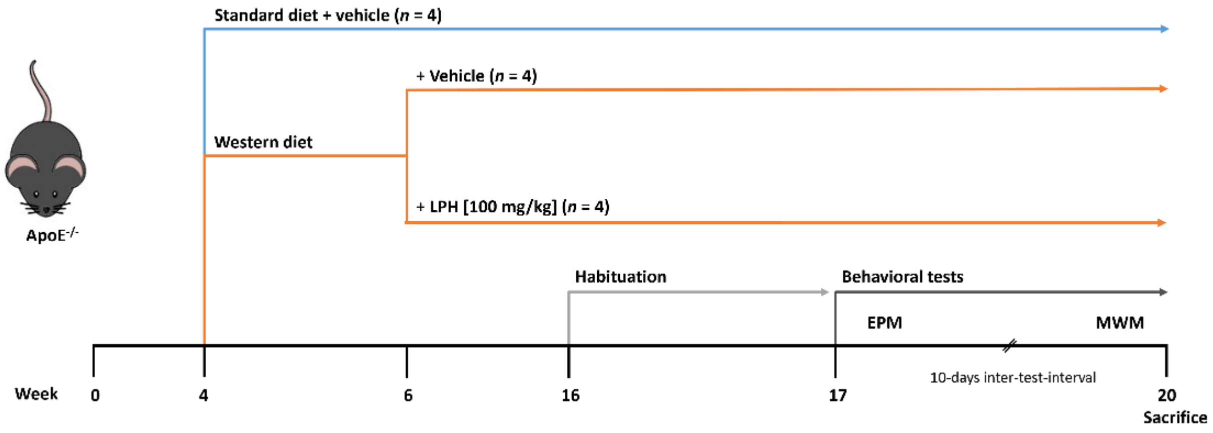

EPM, Elevated Plus Maze; LPH, lupin protein hydrolysate; MWM, Morris Water Maze.

Figure S2. Scheme of the Morris Water Maze protocol.

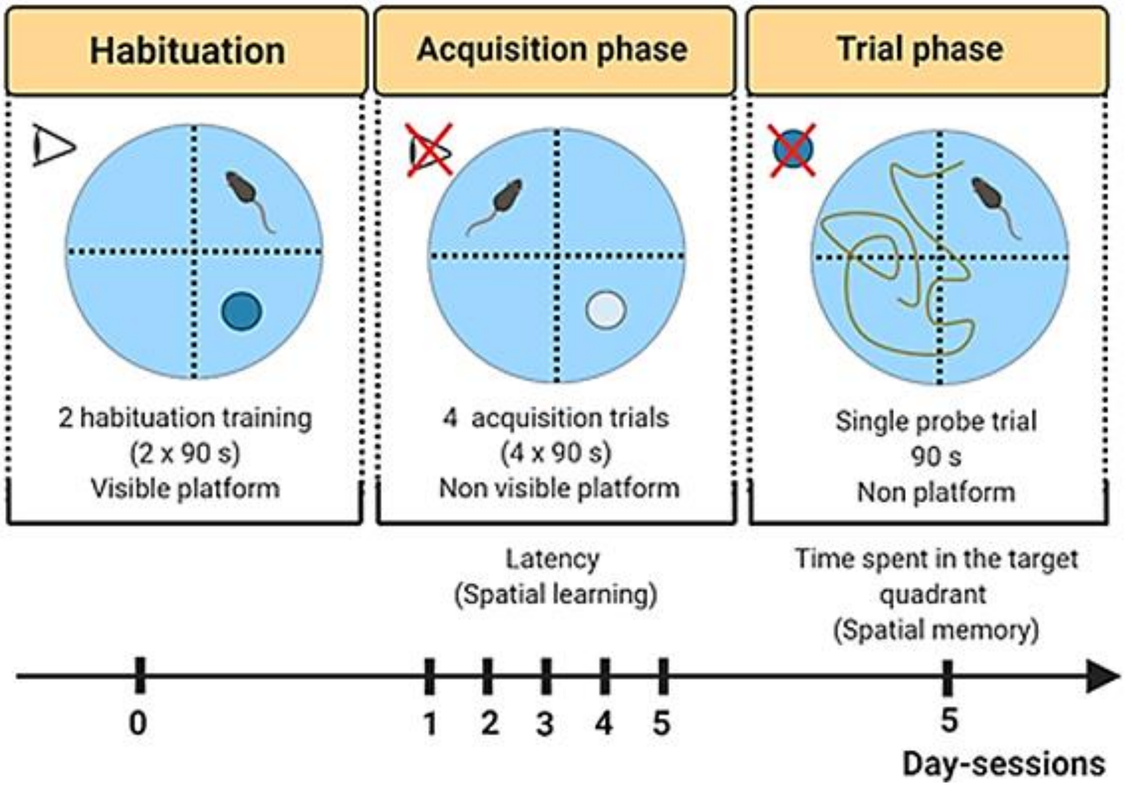

The navy blue circle indicates a visible platform position while the pale blue indicates a hidden platform position. Lines represent imaginal quadrant.

Video material

Video S1. Representative video of standard diet (SD)-fed mice during the elevated plus maze test.

Video S2. Representative video of Western diet (WD)-fed mice during the elevated plus maze test.

Video S3. Representative video of Western diet (WD)-fed and lupin protein hydrolysate (LPH)-treated mice during the elevated plus maze test.

Video S4. Representative video of standard diet (SD)-fed mice during the trial phase of the Morris water maze test.

Video S5. Representative video of Western diet (WD)-fed mice during the trial phase of the Morris water maze test.

Video S6. Representative video of Western diet (WD)-fed and lupin protein hydrolysate (LPH)-treated mice during the trial phase of the Morris water maze test.

References

1. ENVIGO. Teklad Global 14% Protein Rodent Maintenance Diet. Availabe online: https://insights.envigo.com/hubfs/resources/data-sheets/2014s-datasheet-0915.pdf
2. TestDiet. 58V8 - 45 kcal % Fat. Availabe online: https://www.testdiet.com/Diets/High-Fat-DIO/index.html
